# Editorial: Regulated cell death and neurological diseases

**DOI:** 10.3389/fnins.2026.1842251

**Published:** 2026-05-20

**Authors:** Hongjian Pu

**Affiliations:** Department of Neurology, University of Pittsburgh, Pittsburgh, PA, United States

**Keywords:** amyotrophic lateral sclerosis (ALS), apoptosis, autophagy, ferroptosis, inflammation associated death, necroptosis, regulated cell death (RCD)

Amyotrophic lateral sclerosis (ALS) is a fatal neurodegenerative disorder characterized by progressive degeneration of upper and lower motor neurons, leading to paralysis and premature death. Despite sustained research efforts, effective disease-modifying therapies remain limited (Hardiman et al., 2017). While biological heterogeneity and mechanistic complexity are often invoked to describe ALS, these features alone do not necessarily explain the persistent challenges in therapeutic development. An alternative and complementary possibility is that key pathogenic processes—particularly those not fitting traditional frameworks—have not been fully recognized or adequately prioritized.

Increasing evidence indicates that motor neuron loss in ALS does not arise from a single dominant mechanism but reflects the involvement of multiple regulated cell death (RCD) programs (Moujalled et al., 2021). Importantly, these are not limited to classical apoptosis, necroptosis, pyroptosis, or autophagy-associated cell death. Emerging evidence implicates additional modalities such as ferroptosis, parthanatos, lysosomal membrane permeabilization, mitochondrial permeability catastrophe, and other metabolically driven or immunogenic forms of cell death. Collectively, these observations underscore that the current taxonomy of cell death remains incomplete and that neurodegeneration likely engages a broader spectrum of lethal programs than is commonly appreciated.

Over the past decade, neurodegeneration research has shifted from viewing neuronal death as a passive endpoint toward understanding it as an actively regulated and context-dependent process shaped by metabolic stress, redox imbalance, iron homeostasis, and immune signaling. Among newly recognized RCD modalities, ferroptosis—an iron-dependent form of lipid peroxidation-driven cell death—has attracted particular attention because of its mechanistic distinctiveness and relevance to neuronal vulnerability (Dixon et al., 2012; Stockwell et al., 2017). Motor neurons, with their high metabolic demands, enriched polyunsaturated membrane lipids, and limited antioxidant buffering capacity, appear particularly susceptible to such non-apoptotic, metabolism-linked death programs (Masaldan et al., 2019). Nevertheless, ferroptosis represents only one component of a broader and still expanding landscape of regulated neuronal death.

This Research Topic brings together four complementary contributions that collectively explore RCD as a convergent theme across neurodegenerative and neurological disorders, with particular relevance to ALS. Given the scope of a Topic comprising a limited number of articles, the aim here is not to integrate all relevant levels—from molecular mechanisms to clinical phenotypes—into a single unified framework. Rather, the goal is to highlight conceptually important directions and mechanistic priorities that may otherwise remain under-represented in disease-centric discussions.

A central contribution in this Topic provides a comprehensive bibliometric and bioinformatic mapping of the research landscape linking ALS with RCD (Zhang, Zhao et al.). This analysis reveals a clear thematic evolution from early emphasis on oxidative stress and apoptosis toward broader recognition of multiple RCD programs, including ferroptosis, necroptosis, pyroptosis, and autophagy-related mechanisms. Importantly, network-level analyses identify shared hub genes and signaling pathways that bridge cell death execution with immune and metabolic regulation, reinforcing the view that neurodegeneration reflects coordinated engagement of interconnected death programs rather than a single terminal pathway.

Complementary insights are provided by transcriptomic and experimental analyses identifying ferroptosis-regulatory networks in neurodegenerative disease (Tang, Wu et al.). Although focused on Alzheimer's disease, this work highlights regulatory architectures in which ferroptosis intersects with immune modulation and metabolic control, illustrating how neuronal susceptibility can emerge from the interaction of multiple stress-responsive pathways. Such findings support the notion that ferroptosis-like processes may coexist and interact with other cell death modalities in ALS and related disorders.

Additional contributions in this Topic extend these concepts to acute neurological injury. One original research article demonstrates that reduced serum iron levels are associated with the *penumbra freezing* phenomenon in acute ischemic stroke patients presenting beyond conventional treatment windows (Zhang, Xu et al.). While the temporal dynamics of stroke differ fundamentally from those of ALS, this study reinforces a principle of broader relevance: iron-associated metabolic signatures may serve as accessible indicators of tissue vulnerability, reflecting engagement of ferroptosis-linked and other metabolism-driven death pathways (Masaldan et al., 2019).

Finally, a comprehensive review of RCD in severe facial paralysis illustrates how apoptosis, necroptosis, ferroptosis, autophagy, and inflammation-associated death programs interact with axonal injury to shape neuronal survival (Tang, Ji et al.). Although focused on peripheral nerve pathology, this work reinforces a trans-diagnostic concept directly applicable to neurodegeneration: neuronal fate is determined by the integration of multiple regulated death mechanisms operating within a dynamic immune-metabolic microenvironment.

Across the four articles, several translational challenges become apparent. First, the diversity of RCD forms implicated in neurodegeneration complicates mechanistic interpretation and biomarker development, particularly when distinct pathways may operate simultaneously or sequentially. Second, while systems-level and bioinformatic approaches increasingly identify candidate pathways, direct validation linking specific cell death mechanisms to sustained neuronal survival remains limited. These gaps highlight the need for careful prioritization rather than premature unification of heterogeneous mechanisms (Hardiman et al., 2017).

Looking forward, the contributions in this Research Topic suggest that progress in ALS and related disorders may depend on embracing—not simplifying—the diversity of RCD programs. Multimodal strategies combining molecular, cellular, and computational approaches may help disentangle overlapping death mechanisms without forcing them into overly narrow classifications. Machine-learning approaches, in particular, may prove valuable in identifying dominant pathogenic patterns across complex datasets (Grollemund et al., 2019).

In summary, this Research Topic highlights RCD as a critical and evolving framework for understanding neurodegeneration. By explicitly acknowledging the plurality of death mechanisms beyond traditional categories, and by resisting premature integration across disparate levels of analysis, the articles presented here aim to stimulate more nuanced and inclusive approaches to the study of neuronal loss.
